# Hyperkalaemia prevalence and dialysis patterns in Chinese patients on haemodialysis: an interim analysis of a prospective cohort study (PRECEDE-K)

**DOI:** 10.1186/s12882-023-03261-8

**Published:** 2023-08-09

**Authors:** Zhaohui Ni, Haijiao Jin, Renhua Lu, Lihong Zhang, Li Yao, Guojian Shao, Li Zuo, Shuguang Qin, Xinzhou Zhang, Qinghong Zhang, Weimin Yu, Qun Luo, Yuqing Ren, Hui Peng, Jie Xiao, Qiongqiong Yang, Qinkai Chen, Yifan Shi, the PRECEDE-K study group

**Affiliations:** 1 Department of Nephrology, Ren Ji Hospital, Shanghai Jiao Tong University School of Medicine, No. 160, Pujian Road, Shanghai, 200127 China; 2 Department of Nephrology, The First Hospital of Hebei Medical University, Shijiazhuang, Hebei China; 3 Department of Nephrology, The First Hospital of China Medical University, Shenyang, Liaoning China; 4 Department of Nephrology, Wenzhou Central Hospital, Wenzhou, Zhejiang China; 5 Department of Nephrology, Peking University People’s Hospital, Beijing, China; 6 Department of Nephrology, Guangzhou First People’s Hospital, Guangzhou, Guangdong China; 7 Department of Nephrology, Shenzhen People’s Hospital, Shenzhen, Guangdong China; 8 Department of Nephrology, Taihe Hospital, Shiyan, Hubei China; 9 Department of Nephrology, Shanxi Bethune Hospital, Taiyuan, Shanxi China; 10 Department of Nephrology, Hwa Mei Hospital, University of Chinese Academy of Sciences, Ningbo, Zhejiang China; 11 Department of Nephrology, Yangquan Coal Industry (Group) General Hospital, Yangquan, Shanxi China; 12 Department of Nephrology, Third Affiliated Hospital of Sun Yat-Sen University, Guangzhou, Guangdong China; 13 Department of Nephrology, The First Affiliated Hospital of Guangzhou Medical University, Guangzhou, Guangdong China; 14 Department of Nephrology, Sun Yat-Sen Memorial Hospital, Sun Yat-Sen University, Guangzhou, Guangdong China; 15 Department of Nephrology, First Affiliated Hospital of Nanchang University, Nanchang, Jiangxi China; 16 Medical Affairs, AstraZeneca Investment China Co, Shanghai, China

**Keywords:** Dialysate potassium, Haemodialysis, Hyperkalaemia, Potassium fluctuation, Potassium gradien

## Abstract

**Background:**

Hyperkalaemia is a known risk factor for cardiac arrhythmia and mortality in patients on haemodialysis. Despite standard adequate haemodialysis, hyperkalaemia is common in patients with end-stage renal disease (ESRD) at interdialytic intervals. Data on hyperkalaemia burden and its effects on dialysis patterns and serum potassium (sK) fluctuations in patients on haemodialysis in China remain limited. The prospective, observational cohort study (PRECEDE-K; NCT04799067) investigated the prevalence, recurrence, and treatment patterns of hyperkalaemia in Chinese patients with ESRD on haemodialysis.

**Methods:**

Six hundred adult patients were consecutively enrolled from 15 secondary and tertiary hospitals in China. In this interim analysis, we report the baseline characteristics of the cohort, the prevalence of predialysis hyperkalaemia (sK > 5.0 mmol/L), and the trends in serum–dialysate potassium gradient and intradialytic sK shift at Visit 1 (following a long interdialytic interval [LIDI]).

**Results:**

At baseline, most patients (85.6%) received three-times weekly dialysis; mean duration was 4.0 h. Mean urea reduction ratio was 68.0% and Kt/V was 1.45; 60.0% of patients had prior hyperkalaemia (previous 6 months). At Visit 1, mean predialysis sK was 4.83 mmol/L, and 39.6% of patients had hyperkalaemia. Most patients (97.7%) received a dialysate potassium concentration of 2.0 mmol/L. The serum–dialysate potassium gradient was greater than 3 mmol/L for over 40% of the cohort (1– < 2, 2– < 3, 3– < 4, and ≥ 4 mmol/L in 13.6%, 45.1%, 35.7%, and 5.2% of patients, respectively; mean: 2.8 mmol/L). The intradialytic sK reduction was 1– < 3 mmol/L for most patients (0– < 1, 1– < 2, 2– < 3, and ≥ 3 mmol/L in 24.2%, 62.2%, 12.8%, and 0.9% of patients, respectively; mean: 1.4 mmol/L).

**Conclusions:**

Hyperkalaemia after a LIDI was common in this real-world cohort of Chinese patients despite standard adequate haemodialysis, and led to large serum–dialysate potassium gradients and intradialytic sK shifts. Previous studies have shown hyperkalaemia and sK fluctuations are highly correlated with poor prognosis. Effective potassium-lowering treatments should be evaluated for the improvement of long-term prognosis through the control of hyperkalaemia and sK fluctuations.

**Trial registration:**

ClinicalTrials.gov, NCT04799067.

**Supplementary Information:**

The online version contains supplementary material available at 10.1186/s12882-023-03261-8.

## Introduction

End-stage renal disease (ESRD) is the terminal stage of chronic kidney disease (CKD) characterized by glomerular filtration rate < 15 mL/min/1.73m^2^, or the requirement for renal replacement therapy (RRT) [1]. ESRD leads to premature mortality and is recognized as a global public health priority [2]. With a rising ESRD prevalence, RRT use is projected to rise from 3.9 million in 2017 to 5.4 million by 2030, with the largest increase occurring in Asia [3, 4]. Haemodialysis (HD) is the dominant RRT modality worldwide [5, 6].

Hyperkalaemia, defined as elevated serum potassium (sK) levels, is a common complication among patients with ESRD, partly due to their diminished ability for renal potassium excretion [1, 7]. Despite the removal of excess sK with HD treatment, 38–74% of patients with ESRD continued to have persistent hyperkalaemia during HD intervals [8–11]. Hyperkalaemia has been associated with adverse clinical outcomes, including significant arrhythmia, hospitalization, and all-cause mortality [10, 12–15]. Furthermore, together with the indication for low potassium dialysate during HD, hyperkalaemia leads to a steep serum–dialysate potassium gradient, which can bring about large sK fluctuations, potentially triggering cardiac arrhythmia and sudden death [13]. It is hence crucial to recognize the risks of hyperkalaemia and the importance of achieving long-term stable control of sK in patients with ESRD on HD.

In China, the prevalence of ESRD is projected to reach 1505 patients per million population by 2025 [16]. In 2021, approximately 750 000 Chinese patients with ESRD received HD treatment [17]. Chinese patients with ESRD may show distinct trends of hyperkalaemia prevalence and recurrence compared with those in other countries, due to differences in CKD aetiologies, diet, and treatment patterns [18, 19]. The high burden of hyperkalaemia in Chinese patients on HD has been previously reported [10, 20]. To our knowledge, studies on hyperkalaemia prevalence and dialysis patterns in patients on HD in China remain limited, and updated clinical guidelines are lacking [21]. The PRECEDE-K study (ClinicalTrials.gov Identifier: NCT04799067; registered on 16/03/2021) aimed at understanding the prevalence, recurrence, and treatment patterns of hyperkalaemia in Chinese patients with ESRD on HD. In this interim analysis, we report the cohort characteristics at enrolment, as well as hyperkalaemia prevalence, serum–dialysate potassium gradient, and sK fluctuation patterns at the baseline HD visit, hereon referred to as Visit 1.

## Methods

### Study design and patients

The protocol of this prospective cohort study has been previously published [22]. Briefly, patients aged ≥ 18 years with ESRD who were on HD treatment were consecutively enrolled from 15 HD centres in secondary and tertiary hospitals in China. Key exclusion criteria included acute kidney injury, ongoing peritoneal dialysis usage, and expected renal transplantation in the next 6 months.

This study was conducted in accordance with the Declaration of Helsinki, the International Conference on Harmonization’s Guidelines for Good Clinical Practice, and applicable local legislation on non-interventional and/or observational studies. All patients provided written and oral informed consent pre-enrolment. The protocol and all its amendments were approved by the Shanghai Jiao Tong University School of Medicine Renji Hospital Ethics Committee (2020–040) and the ethics committee of each participating centre. The study followed the Strengthening the Reporting of Observational Studies in Epidemiology (STROBE) reporting guidelines.

### Procedures

At Visit 1, patients were in a long (≥ 2-day) interdialytic interval (LIDI) of the HD cycles. Baseline characteristics collected included demographics, medical history, ESRD aetiology, concomitant medications, dialysis vintage, pre- and postdialysis sK levels, and other laboratory measurements.

Patients were followed up every 4 weeks up to 24 weeks, or until death, loss to follow-up, change of RRT modality, withdrawal of informed consent, or termination as deemed necessary by investigators, whichever occurred earlier. At each follow-up visit after a LIDI, data collected included predialysis sK measurements (postdialysis sK measurements were not mandatory beyond Visit 1), dialysis parameters (i.e., dialysis adequacy, the prescribed duration of dialysis, and dialysate potassium concentration), concomitant medications, and other clinical and laboratory findings. While twice weekly HD is characterized by two LIDIs each week, three-times weekly HD leads to two short (1-day) interdialytic intervals (SIDIs), in addition to one LIDI. Thus, patients who received three-times weekly HD treatments were indicated for an additional follow-up visit after a SIDI (at Day 3 or 5) for sK analysis.

### Outcomes

The primary endpoint was the proportion of patients who experienced any hyperkalaemia event (defined as sK > 5.0 mmol/L [23, 24]) at the study enrolment, or during follow-up. Secondary endpoints included intradialytic potassium shift (defined as the difference between pre- and postdialysis sK levels) following a LIDI during the first week after enrolment. This interim analysis reports the prevalence of predialysis hyperkalaemia after a LIDI, the serum–dialysate potassium gradient, and the intradialytic potassium shift at Visit 1.

### Sample size estimations

According to previous reports, 58.0% of patients on HD experienced hyperkalaemia during a 4-month follow-up period [10], and 73.8% over 2 years [11]. Thus, it was assumed that 58.0–73.8% of patients would develop hyperkalaemia during this 24-week study. A sample size of 600 patients was accordingly planned to provide a clinically acceptable precision estimate of 3.5–3.9% for the primary endpoint.

### Statistical analysis

The full analysis set (FAS), defined as all enrolled patients, was used for this interim analysis. Data were presented as descriptive statistics. Categorical data were shown as number and percentages. Continuous variables, including pre- and postdialysis sK, were presented as means with standard deviations, and medians with ranges.

## Results

### Patient disposition and baseline characteristics

**Fig. 1:**
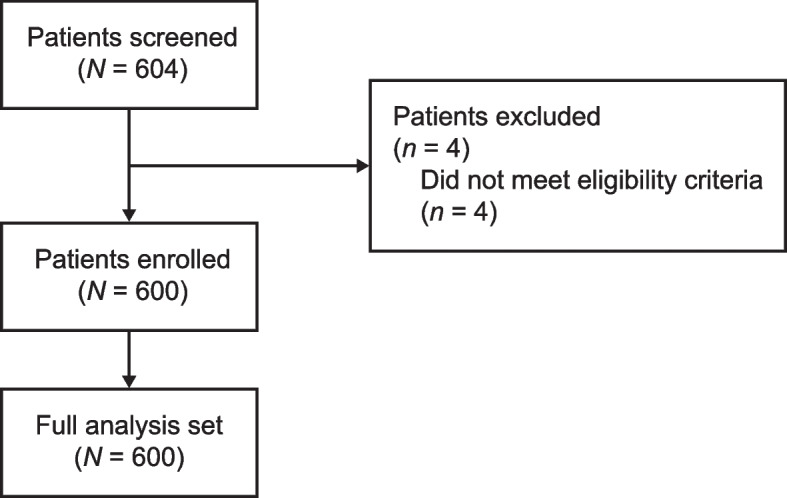
Subject disposition

Among 604 patients screened, 600 met the eligibility criteria and enrolled into the FAS (Fig. 1). At the time of this interim analysis (Visit 1), four patients had missing pre-dialysis sK measurements and nine had missing post-dialysis sK measurements, but all patients remained on the study. At baseline, the median age was 55.0 years, and 403 patients (67.2%) were male (Table 1). The most common causes of ESRD were primary glomerulonephritis (188 [38.8%]), diabetic kidney disease (135 [27.8%]), and hypertensive renal disease (77 [15.9%]).

**Table 1:** Baseline characteristics in the FAS

Parameter	FAS^a^ (*N* = 600)
**Demographics**	
Age, years	
Mean (SD)	54.3 (12.9)
Median (IQR)	55.0 (45.0–63.5)
Sex, *n* (%)	
Male	403 (67.2)
**Disease characteristics and concomitant conditions**	
Aetiology of ESRD, *n* (%)	
Primary glomerulonephritis	188 (38.8)
Diabetic kidney disease	135 (27.8)
Hypertensive renal disease	77 (15.9)
Any medical history^b^, *n* (%)	599 (99.8)
Hyperkalaemia	360 (60.0)
Renal anaemia	487 (81.2)
Hypertension	487 (81.2)
Hyperphosphataemia	277 (46.2)
Diabetes mellitus	119 (19.8)
Metabolic acidosis	79 (13.2)
Hyperlipidaemia	75 (12.5)
Coronary artery disease	75 (12.5)
Hyperuricaemia	61 (10.2)
**Dialysis parameters**	
Type of haemodialysis, *n* (%)	
Haemodialysis	529 (88.3)
Haemodiafiltration	60 (10.0)
Haemoperfusion combined with haemodialysis or haemodiafiltration	10 (1.7)
Vascular access, *n* (%)	
Arteriovenous fistula	568 (94.8)
Central tunnelled dialysis catheter	14 (2.3)
Arteriovenous graft	9 (1.5)
Other (temporary catheter)	8 (1.3)
Dialysis frequency, *n* (%)	
Three-times weekly	513 (85.6)
Five times every 2 weeks	50 (8.3)
Twice weekly	36 (6.0)
Dialysate potassium concentration, mmol/L	
Mean (SD)	2.0 (0.14)
Median (IQR)^c^	2.0 (2.0–2.0)
Haemodialysis duration, hours	
Mean (SD)	4.0 (0.16)

*ESRD* end-stage renal disease, *FAS* full analysis set, *IQR* interquartile range, *SD* standard deviation

^a^Aetiology of ESRD (*n* = 485; 115 missing); medical history and all dialysis parameters (*n* = 599; one missing)

^c^Range: 2.0–3.0 mmol/L

The most common type of HD used for ESRD treatment was conventional HD alone (529 [88.3%]), followed by haemodiafiltration (60 [10.0%]), and haemoperfusion combined with HD or haemodiafiltration (10 [1.7%]; Table 1). The mean dialysis duration was 4.0 h. Most patients (513 [85.6%]) received dialysis three-times weekly, while 36 (6.0%) and 50 (8.3%) patients received dialysis twice weekly and five times every 2 weeks, respectively.

Nearly all patients (599 [99.8%]) had a medical history (Table 1); 360 patients (60.0%) had prior hyperkalaemia in the past 6 months. Common prior conditions included hypertension (487 [81.2%]), renal anaemia (487 [81.2%]), and hyperphosphataemia (277 [46.2%]). Additional baseline characteristics are shown in Supplementary Table 1.

### Prevalence of predialysis hyperkalaemia

**Fig. 2:**
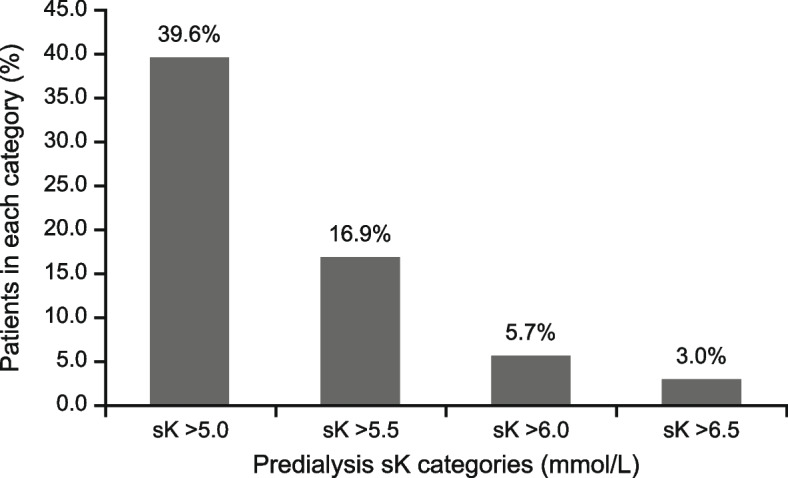
Predialysis serum potassium (sK) levels in the full analysis set (*n* = 596; four missing) at Visit 1

At Visit 1, the mean predialysis sK after the LIDI was 4.83 mmol/L (Table 2), and 236 patients (39.6%) had hyperkalaemia (Fig. 2). The proportions of patients with sK > 5.5, > 6.0, and > 6.5 mmol/L were 101 (16.9%), 34 (5.7%), and 18 (3.0%), respectively.

**Table 2:** Pre- and postdialysis serum potassium in the FAS at Visit 1

sK	FAS (*N* = 600)^a^
Predialysis, mmol/L	
Mean (SD)	4.83 (0.76)
Median (IQR)	4.80 (4.30–5.29)
Range	2.90–7.90
Postdialysis, mmol/L	
Mean (SD)	3.48 (0.549)
Median (IQR)	3.43 (3.15–3.72)
Range	1.70–6.70

*FAS* full analysis set, *IQR* interquartile range, *SD* standard deviation, *sK* serum potassium

^**a**^Predialysis sK (*n* = 596; four missing); postdialysis sK (*n* = 591, nine missing)

### Serum–dialysate potassium gradient and other dialysis parameters

**Fig. 3:**
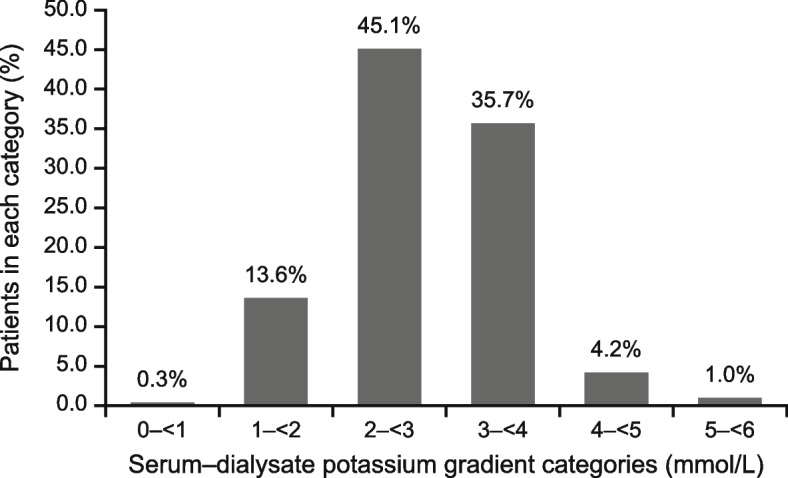
Serum–dialysate potassium gradient in the full analysis set (*n* = 596; four missing) at Visit 1

At Visit 1, most patients (585 [97.7%]) were prescribed a dialysate potassium concentration of 2.0 mmol/L, while 2.5 mmol/L and 3.0 mmol/L dialysate potassium were used in three (0.5%) and 11 (1.8%) patients, respectively (Table 3). The mean serum–dialysate potassium gradient was 2.8 mmol/L. The proportions of patients who had serum–dialysate potassium gradients of 0– < 1, 1– < 2, 2– < 3, 3– < 4, and ≥ 4 mmol/L were 0.3%, 13.6%, 45.1%, 35.7%, and 5.2%, respectively (Fig. 3).

**Table 3:** Dialysis parameters in the FAS at Visit 1

Dialysis parameters	FAS (*N* = 600)^a^
Dialysate potassium concentration (mmol/L), *n* (%)	
2.0	585 (97.7)
2.5	3 (0.5)
3.0	11 (1.8)
Haemodialysis duration (hours), *n* (%)	
3	12 (2.0)
4	583 (97.3)
5	4 (0.7)
URR (%)	
Mean (SD)	68.0 (9.70)
Kt/V	
Mean (SD)	1.45 (0.496)

*FAS* full analysis set, *SD* standard deviation, *URR* urea reduction ratio

^**a**^Dialysate potassium and haemodialysis duration (*n* = 599; one missing); URR (*n* = 589, 11 missing); Kt/V (*n* = 577, 23 missing)

Most patients (583 [97.3%]) were prescribed 4 h of dialysis at Visit 1; this ranged from 3–5 h (Table 3). The mean urea reduction ratio (URR) was 68.0% and urea clearance as measured by Kt/V was 1.45. The standard for dialysis adequacy, indicated by URR > 65% and Kt/V > 1.2, was achieved by 381 (64.7%) and 434 (75.2%) patients, respectively.

### Postdialysis sK and intradialytic sK shift

**Fig. 4:**
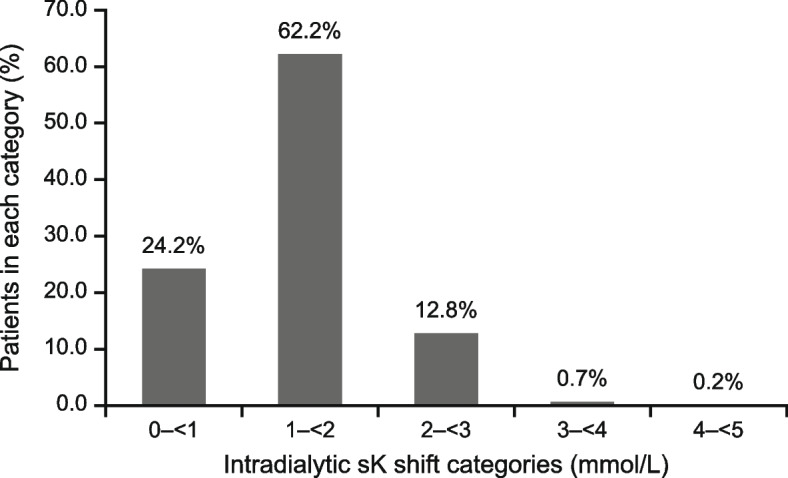
Intradialytic serum potassium (sK) shift in the full analysis set (*n* = 587; 13 missing) at Visit 1

The mean postdialysis sK at Visit 1 was 3.48 mmol/L (Table 2), yielding a mean intradialytic sK shift of − 1.35 mmol/L. Intradialytic sK reductions of 0– < 1, 1– < 2, 2– < 3, and ≥ 3 mmol/L were observed in 142 (24.2%), 365 (62.2%), 75 (12.8%), and five (0.9%) patients, respectively (Fig. 4). Only four patients (0.7%) had hyperkalaemia postdialysis (Supplementary Fig. 1).

## Discussion

To our knowledge, PRECEDE-K is the first and largest prospective cohort study to investigate the prevalence of hyperkalaemia and its implications on sK fluctuations during HD, both of which present a potential risk for acute cardiovascular events, in Chinese patients with ESRD. In this interim analysis, we showed that hyperkalaemia was present in 60% of the cohort in the previous 6 months and nearly 40% at Visit 1. A dialysate potassium concentration of 2.0 mmol/L was prescribed for nearly all patients (98%) during the HD session at Visit 1. The resulting serum–dialysate potassium gradient was > 3 mmol/L for over 40% of the cohort, with a mean of 2.8 mmol/L. An intradialytic sK reduction of ≥ 1 mmol/L occured in over 75% of patients (1– < 3 mmol/L in the majority), with a mean of − 1.4 mmol/L.

Owing to the intermittent nature of HD, predialysis hyperkalaemia can arise from the sK rebound during the interdialytic period [25, 26]. While extracellular potassium ions are rapidly depleted during HD, an accelerated shift of potassium ions from the intracellular to extracellular space occurs during the interdialytic period to restore the balance between the two compartments. Approximately 40% of the cohort had predialysis hyperkalaemia at Visit 1 (following a LIDI), which was moderate-to-severe (sK > 5.5 mmol/L) in over 25% of patients. The high burden of predialysis hyperkalaemia was similarly reported in Chinese and global studies. A global prospective study showed that for Chinese patients on maintenance HD, predialysis hyperkalaemia occurred in 75.0% of the 4-monthly periods evaluated [10]. In a retrospective cohort of Chinese patients on HD, 63% had hyperkalaemia, among whom 65% experienced recurrent episodes [20]. A prospective study in France showed that 73.8% of patients on HD had predialysis hyperkalaemia (sK > 5.1 mmol/L) over 2 years [11]. In a retrospective observational cohort of patients on HD in the United States (US), 74% experienced predialysis hyperkalaemia (sK > 5.0 mEq/L) within 1 year, 52% within 3 months, and 38% within 1 month [8]. Another US database study showed that the prevalence of sK ≥ 5.5 mEq/L the day after a LIDI was 2.0–2.4 times higher than that the day after a SIDI [12]. sK at SIDI versus that at LIDI in a subset of the cohort who received HD three-times weekly, as well as the occurrence and recurrence of hyperkalaemia over the 24-week follow-up period, are other prespecified outcomes to be reported in the PRECEDE-K study [22]. Furthermore, potential risk factors of hyperkalaemia occurrence and recurrence, including but not limited to dialysis adequacy, dialysis frequency, dialysate potassium concentration, dialysis vintage, medical history of special interest, and treatment of hyperkalaemia, will be analysed as exploratory endpoints [22].

Hyperkalaemia was associated with increased risks of arrythmia events, hospitalizations, and mortality in patients with ESRD on HD [9, 10, 12, 13, 27–30]. The risk of sudden cardiac arrest increased by 38% for each 1 mEq/L increase in predialysis sK above 5.1 mEq/L [30]. Compared with patients on HD with predialysis sK < 5.1 mEq/L, the risks of all-cause mortality over 4 months were 15%, 19%, and 33% higher in those with predialysis sK 5.1–5.5, 5.6–6.0, and > 6.0 mEq/L, respectively [10]. Similar increased risks of all-cause hospitalization, and the composite of cardiovascular death or hospitalization, with hyperkalaemia were observed [10].

The steepness of the serum–dialysate potassium gradient, which depends on both the dialysate potassium concentration and predialysis sK, determines the extent of intradialytic sK shift. In the PRECEDE-K cohort, a uniform dialysate potassium concentration of 2.0 mmol/L was most used, this led to steep serum–dialysate potassium gradients (> 3 mmol/L for over 40% of the cohort), and in turn large intradialytic sK shifts (≥ 1 mmol/L for over 75% of patients), particularly in those with high predialysis sK levels. A global study similarly reported the common use of 2.0 mEq/L dialysate potassium prescription in several countries, including China (in 84% of the facilities) [13]. Dialysate potassium concentrations of 2.0–2.5 mmol/L was most used worldwide, although large variations in dialysate potassium prescriptions existed [13, 31, 32]. Among the serum–dialysate potassium gradients recorded from a large dialysis organization in the US, approximately 40% were ≥ 3 mmol/L [31], consistent with our findings.

Nevertheless, optimizing the dialysate potassium concentration to control the extent of intradialytic sK removal remains challenging. High dialysate potassium concentrations result in gradual serum–dialysate potassium gradients and low intradialytic sK shifts, which may be insufficient to manage hyperkalaemia and its associated risks [31, 33]. Low dialysate potassium concentrations result in steep serum–dialysate potassium gradients, which may correct hyperkalaemia, albeit with associated cardiovascular risks due to the accompanying large intradialytic sK shifts [31, 33]. Indeed, the association between low potassium dialysate baths and clinical outcomes, mainly sudden cardiac death and all-cause mortality, was observed to be contradictory. A low potassium (1 mEq/L) dialysate bath decreased the risks of sudden cardiac arrest or death through correcting hyperkalaemia [34–36]. In contrast, other available evidence demonstrated increased risks for malignant arrythmia and other acute cardiovascular events with the use of low dialysate potassium concentrations that led to steep serum–dialysate potassium gradients, particularly in patients with high predialysis sK [33]. Dialysate potassium concentrations < 3 mEq/L were potentially associated with higher risk of sudden death [37]. Patients with cardiac arrest during HD were more likely to be receiving serum dialysate potassium concentration < 2 mEq/L [30, 38]. It was suggested that increased mortality with low dialysate potassium concentrations may be dependent on predialysis sK, as observed in patients with sK ≥ 5 mEq/L but not in those with sK < 5 mEq/L, possibly due to the larger serum–dialysate potassium gradients in the former group [39]. Serum–dialysate potassium gradient ≥ 3 mEq/L was independently associated with increased risks of hospitalizations and emergency department visits [31]. Patients with sK fluctuations > 1 mmol/L during HD showed significantly increased rates of malignant arrhythmia [40], which can be attributable to increased intradialytic cell membrane polarization [26]. A greater intradialytic potassium shift has also been associated with a more rapid sK rebound postdialysis [25, 31, 33, 41]; this can contribute to predialysis hyperkalaemia and the associated worsened clinical outcomes in a concatenation of events. These findings highlight the limitations in managing hyperkalaemia through the optimization of dialysate potassium concentrations.

Taken together, our findings support the high prevalence of predialysis hyperkalaemia following an LIDI in Chinese patients with ESRD on HD treatment. Predialysis hyperkalaemia, together with the use of a uniform dialysate potassium concentration, led to steep serum–dialysate potassium gradients (≥ 3 mEq/L) and resulting large intradialytic sK fluctuations (> 1 mmol/L) in substantial proportions of patients in the PRECEDE-K cohort; and these are established risk factors for acute cardiovascular events and death [9, 10, 12, 13, 27–31, 33, 35, 37–40].

Effective management of hyperkalaemia in patients on HD during the interdialytic period, particularly at LIDIs, is needed. A recent consensus guideline provided recommendations on hyperkalaemia management in the HD setting, including the use of potassium binders [21]. Novel potassium binders, namely sodium zirconium cyclosilicate (SZC) and patiromer, were recently approved for the treatment of chronic or recurrent hyperkalaemia [42, 43]. In China, SZC is currently the only novel potassium binder available for hyperkalaemia management and has been approved for use in the chronic HD setting based on the global Phase 3b DIALIZE study (NCT03303521) [44]. Among patients with three-times weekly HD and predialysis hyperkalaemia in the DIALIZE study, SZC treatment once daily on nondialysis days versus placebo resulted in a significantly higher proportion of patients who maintained sK 4.0–5.0 mmol/L in at least three of four LIDIs without the need for rescue therapy (41.2% versus 1.0%; odds ratio, 68.8 [95% confidence interval, 10.9–2810.9]; *p* < 0.001) [45]. SZC lowered pre- and postdialysis mean sK levels and maintained them at steady levels over 8 weeks of treatment [45]. In contrast, patients in the placebo arm experienced sK fluctuations between dialysis and nondialysis days [45]. With lower predialysis sK levels, patients on SZC treatment achieved a mean reduction of 0.74 mmol/L in their serum–dialysate potassium gradient and a shift towards lower-risk potassium gradient categories, without the need for any dialysate potassium concentration changes, compared with those on placebo [46]. These results were corroborated by the DIALIZE China trial (NCT04217590). Additional outcomes of the PRECEDE-K study, including the proportions of patients with hyperkalaemia or such events treated with potassium binders over 24 weeks, will inform the specific treatment patterns of Chinese patients with ESRD in the HD setting [22].

### Limitations

First, the results of this interim analysis were for a single time point (Visit 1). However, as patients were already on HD prior to enrolment, the observations at this visit may be representative of the outcomes following a LIDI over the course of maintenance HD; these outcomes will be further evaluated over 24 weeks. Second, this study did not report the association between hyperkalaemia and prognosis. Predialysis hyperkalaemia, steep serum–dialysate potassium gradients, and large intradialytic sK shifts were previously associated with worse clinical outcomes [9, 10, 12, 13, 27–31, 33, 35, 37–40], although direct causality is yet to be determined. Third, it remains to be determined whether hyperkalaemia observed following the LIDI is due to sK rebound that is associated with steep serum–dialysate potassium gradients and large intradialytic sK shifts. Lastly, the comparison of serum and plasma potassium concentrations to rule out any potential pseudohyperkalaemia was not performed.

## Conclusion

Previous studies have demonstrated that hyperkalaemia and sK fluctuations are highly correlated with poor prognosis in patients on HD. We report that hyperkalaemia and sK fluctuations remain common in Chinese patients with ESRD despite standard adequate HD, which is related to the intermittent nature of potassium removal due to the interdialytic periods. As demonstrated in the DIALIZE trial, potassium-lowering treatment on nondialysis days may control for hyperkalaemia and limit sK fluctuations during LIDIs. Further studies on the effectiveness of potassium-lowering treatments in preventing acute cardiac events and improving long-term prognosis in this setting are warranted.

### Supplementary Information

**Additional file 1:** **Supplementary Table 1.** Other baseline characteristics in the FAS. **Supplementary Fig. 1.** Postdialysis serum potassium (sK) levels in the full analysis set (*n* = 591; nine missing) at Visit 1.

## Data Availability

All data generated or analysed during this study are included in this published article and its supplementary material. Any additional data underlying this article are available from the corresponding author on reasonable request.

## Abbreviations

CKD: Chronic kidney disease
ESRD: End-stage renal disease
FAS: Full analysis set
HD: Haemodialysis
IQR: Interquartile range
LIDI: Long interdialytic interval
RRT: Renal replacement therapy
SD: Standard deviation
SIDI: Short interdialytic interval
sK: Serum potassium
SZC: Sodium zirconium cyclosilicate
URR: Urea reduction ratio
US: United States
