# Supplementary material for: Hyperkalaemia prevalence and dialysis patterns in Chinese patients on haemodialysis: an interim analysis of a prospective cohort study (PRECEDE-K)
Source: BMC Nephrol. 2023 Aug 9;24:233. doi: 10.1186/s12882-023-03261-8 (PMC10411008; doi:10.1186/s12882-023-03261-8)
Supplement: Supplementary file 1 — Additional file 1: Supplementary Table 1. Other baseline characteristics in the FAS. Supplementary Fig. 1. Postdialysis serum potassium (sK) levels in the full analysis set (n = 591; nine missing) at Visit 1. [file 12882_2023_3261_MOESM1_ESM.docx]

**Hyperkalaemia prevalence and dialysis patterns in Chinese patients on haemodialysis: an interim analysis of a prospective cohort study (PRECEDE-K)**

# Supplementary Materials

### **Supplementary Table 1** Other baseline characteristics in the FAS

| **Parameter** | **FAS**^a^ **(*N* = 600)** |
| --- | --- |
| Race, *n* (%) |  |
| Asian | 600 (100) |
| Height, cm |  |
| Mean (SD) | 166.2 (7.85) |
| Median (IQR) | 167.0 (160.0–172.0) |
| Range | 138.0–186.0 |
| Predialysis weight, kg |  |
| Mean (SD) | 65.5 (13.07) |
| Median (IQR) | 64.2 (56.0–73.8) |
| Range | 34.6–125.3 |
| Postdialysis weight, kg |  |
| Mean (SD) | 62.6 (12.77) |
| Median (IQR) | 61.4 (53.5–70.4) |
| Range | 31.9–122.0 |
| Body mass index^b^, kg/m^2^ |  |
| Mean (SD) | 23.6 (3.76) |
| Median (IQR) | 23.4 (20.9–26.0) |
| Range | 15.3–40.9 |

*FAS* full analysis set, *IQR* interquartile range, *SD* standard deviation

^a^ Height (*n* = 599; one missing); predialysis weight (*n* = 598, two missing); postdialysis weight (*n* = 597, three missing); body mass index (*n* = 598; two missing). ^b^ Calculated using predialysis body weight


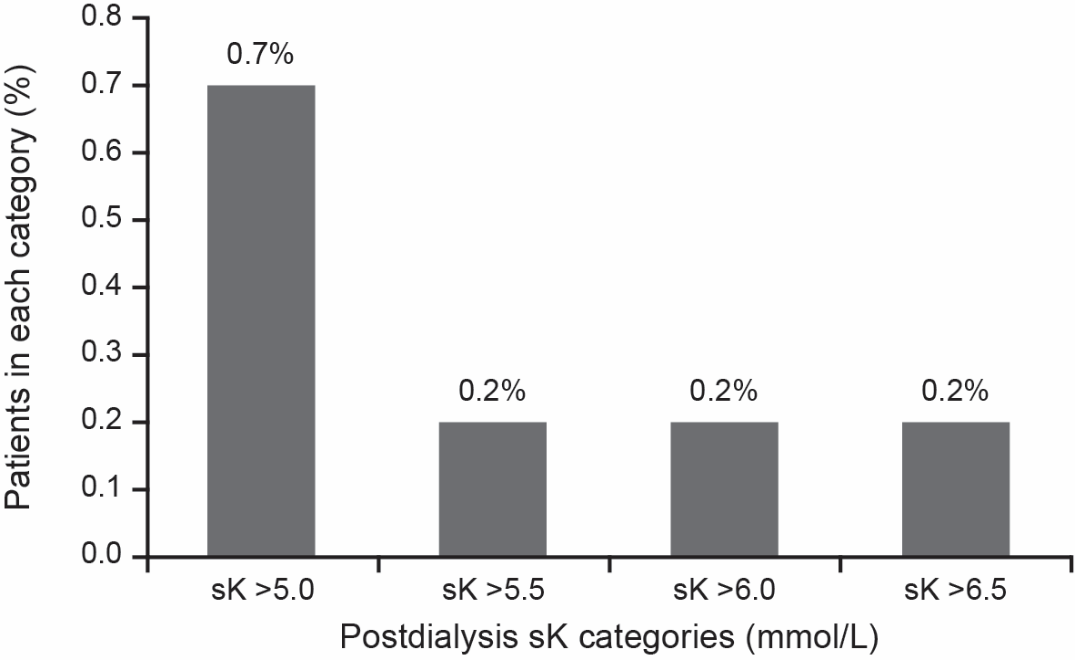


### **Supplementary Fig. 1** Postdialysis serum potassium (sK) levels in the full analysis set (*n* = 591; nine missing) at Visit 1
